# The Role of Health Literacy in Health Behavior, Health Service Use, Health Outcomes, and Empowerment in Pediatric Patients with Chronic Disease: A Systematic Review

**DOI:** 10.3390/ijerph182312464

**Published:** 2021-11-26

**Authors:** Lisa Riemann, Johanna Sophie Lubasch, Axel Heep, Lena Ansmann

**Affiliations:** 1 Organizational Health Services Research, School of Medicine and Health Sciences, University of Oldenburg, Ammerlaender Heerstr. 114-118, 26129 Oldenburg, Germany; lisa.riemann@uol.de (L.R.); lena.ansmann@uol.de (L.A.); 2 Department of Paediatrics, School of Medicine and Health Sciences, University of Oldenburg, Rahel-Straus-Str. 10, 26133 Oldenburg, Germany; axel.heep@uol.de

**Keywords:** health literacy, children, adolescents, review, chronic conditions, health outcomes, health behavior, empowerment

## Abstract

About 8% of all children and adolescents worldwide are affected by chronic diseases. Managing chronic conditions requires pediatric patients to be health literate. The purpose of this review is to examine the existing evidence on the links between health literacy and its outcomes proposed by the model by Sørensen et al. in chronically ill pediatric patients. Four electronic databases (PubMed, Scopus, CINAHL, PsycINFO) were searched to identify pertinent articles published up to November 2021. The search was conducted independently by two researchers and restricted to observational studies. Of 11,137 initial results, 11 articles met eligibility criteria. Overall, 6 studies identified a significant association between health literacy and one of the considered outcomes. Regarding health behavior, none of the studies on adherence found significant associations with health literacy. The results in terms of health service use were inconclusive. Regarding health outcomes, health literacy did not affect most physiological parameters, but it significantly improved health-related quality of life. Overall, evidence remains inconclusive but suggests that health literacy is associated with self-efficacy, health-related quality of life, and health service use in pediatric patients. Further research should be undertaken to strengthen the evidence.

## 1. Introduction

Since the last century, the epidemiology of relevant pediatric diseases has changed significantly [1]. While infectious diseases have declined, chronic diseases in childhood have become prevalent [2]. While in 1960, 1.8% of all children worldwide suffered from chronic conditions, this number rose to 8% by 2010 [3,4,5]. The most prevalent chronic diseases include asthma, obesity, diabetes, as well as mental illness and developmental disabilities [4]. Continued medical advances have resulted in improved long-term prognoses and higher survival rates in children with common diseases, like cancer, cystic fibrosis (CF), or renal failure [1,6,7]. Living with a chronic disease in childhood as well as treatment side effects impact children’s wellbeing in many ways. Besides suffering the resulting physical damage, chronically ill children are often reported to experience traumatic situations in inpatient care [8,9]. Considering that childhood and adolescence are crucial developmental phases characterized by important physical, emotional, cognitive, and behavioral changes [10], chronic disease represents an additional challenge and can consequently lead to social, emotional, developmental, and psychological difficulties [6,8,9,11,12]. In recent years, a growing number of studies have explored late consequences of chronic childhood conditions [6]. Recent studies have demonstrated an association between chronic pediatric somatic diseases and a higher rate of psychological difficulties in adulthood, especially anxiety and depression [9,11,13]. Besides, children with common chronic diseases achieve a lower overall health-related quality of life (HRQoL) [14,15]. Moreover, chronic conditions in particular require a high level of individual responsibility for managing the disease and participating in its treatment [16]. In this regard, pediatric patients often face challenges, including consulting with specialists, adhering to medication, and adjusting their everyday life to their chronic disease [16,17]. 

It has been stressed that in order to prevent adverse long-term effects, pediatric patients themselves need to take an active role alongside their caregiver’s responsibility. This becomes increasingly important in the course of adolescence and becomes indispensable in the context of transition. Transition readiness is defined as “the purposeful, planned movement of adolescents and young adults with chronic physical and medical conditions from child-centered to adult-oriented health care systems” [18]. To strengthen skills related to self-management and coping, patients need to be able to adequately comprehend and implement relevant instructions [19,20]. For this purpose, pediatric patients’ health literacy may be particularly important. Health literacy has been defined as ”the degree to which individuals have the capacity to obtain, process, and understand basic health information and services needed to make appropriate health decisions“ [21]. However, there is no common consensus about the definition of health literacy. Based on a systematic review, Sørensen et al. delivered one approach for an integrated definition and an encompassing, conceptualized model of health literacy, incorporating both public health and medical perspectives [22]. This model defines health literacy as a process, including “12 dimensions of health literacy, referring to the competencies related to accessing, understanding, appraising, and applying health information in the domains of healthcare, disease prevention, and health promotion, respectively” [22]. According to Sørensen et al., health literacy influences eight categories of outcomes, including health behavior, health outcomes, health service use, empowerment, participation, health costs, equity, and sustainability [22]. These categories are regarded as potential outcomes of health literacy within our review. The model by Sørensen et al. also conceptualizes personal, situational, societal, and environmental determinants of health literacy [22]. According to Rothmann et al., some additional aspects are specific to child health literacy and have to be taken into account, namely developmental changes in childhood, specific epidemic factors, dependence of children on their families and caregivers, and various socio-demographic characteristics [23].

In recent years, a growing number of studies has established a link between adult health literacy and the above-mentioned conceptual model. In various adult populations, a low level of health literacy is associated with poorer health outcomes and health behaviors, such as disease management skills or medication adherence [24,25,26,27,28]. Research further found significant associations between health literacy and mortality, hospitalization, and laboratory parameters in chronic kidney disease patients [29]. Individual health literacy is affected by various factors, namely gender, age, culture, cognitive skills, and social skills [30]. In adolescents, additional influences such as family and peer groups but also hormonal factors impact health literacy and behavior [10,30,31]. Studies revealed that a low level of health literacy is associated with higher health care costs [32] and greater usage of health services [33,34] and is disproportionally more common in populations with lower educational and social status as well as in people with a migration background [35]. Within these vulnerable populations, chronic diseases are more prevalent [36,37].

While the available evidence on health literacy in the adult population supports a link between health literacy and diverse outcomes [38], this may not apply to children and adolescents. Existing research on children and adolescents has mainly focused on healthy subjects or on the health literacy of pediatric patients’ parents and caregivers [39,40]. Children and adolescents with chronic conditions have so far rarely served as research subjects. Research studying the link between health literacy and tobacco and alcohol use in healthy adolescents has confirmed an adverse association [39,41]. Findings on parents and caregivers suggest a link between their health literacy and pediatric patients’ outcomes. For instance, an inadequate level of parental health literacy was associated with poor glycemic control in children with diabetes [42,43]. Moreover, prior research found a relationship between low levels of parental health literacy and a more severe course of asthma in children [44]. In recent years, a growing number of studies focusing on the benefits of child health literacy on several health outcomes in populations with chronic diseases has been published. A review published on the topic in 2009 showed inconclusive findings on the link between child health literacy and important health outcomes [39]. Further research on this topic is needed, as the review published in 2009 did not focus on children and adolescents with chronic diseases but on healthy children and adolescents only. Moreover, several studies published since 2009 may potentially have generated further evidence. In order to summarize the current state of research, we aim to conduct the first systematic review of the literature with the following research question: What role does health literacy play in children with chronic illness? Which outcomes have been assessed in previous studies? Although an increase in interventional studies can be seen in recent years [38,45,46,47], the general association between health literacy and its consequences in pediatric children with chronic diseases has not yet been described comprehensively. We regard this as a necessary precondition for developing effective interventions. Consequently, this review focuses on observational studies.

## 2. Materials and Methods

The reporting of this review was conducted in accordance with the PRISMA (Preferred Reporting Items for Systematic Review and Meta-Analyses) statement [48].

### 2.1. Data Sources and Search Strategy

The literature search was performed in four electronic databases including PubMed, CINAHL, PsycINFO, and Scopus, from inception to November 2021. To update the results prior to publication, the search strategy was applied once more for evidence published between July and November 2021. Furthermore, reference lists of included publications were screened for additional relevant studies. The algorithm for the database screening process was generated by connecting particular search terms. Search terms were combined with Boolean operators (AND/OR). Search strategies were developed according to the specific requirements of the databases:

- **pubmed**

((“health literacy” [tiab]) AND (“adolescent” [MeSH Terms] OR “child” [MeSH Terms] OR “hospitals, pediatric” [MeSH Terms] OR “minors” [MeSH Terms] OR “pediatrics” [MeSH Terms] OR “pediatric nursing” [MeSH Terms] OR “puberty” [MeSH Terms] OR “schools” [MeSH Terms] OR “students” [MeSH Terms] OR adoles* OR boy [tiab] OR child* [tiab] OR girl* [tiab] OR highschool* [tiab] OR infant* [tiab] OR juvenil* [tiab] OR kids* [tiab] OR teenage* [tiab] OR kindergar* [tiab] OR minors* [tiab] OR paediat* [tiab] OR pediat* [tiab] OR prepuberty* [tiab] OR prepubescen* [tiab] OR preschool* [tiab] OR puber* [tiab] OR pubescen* [tiab] OR school*[tiab] OR teen* [tiab] OR “under age” [tiab] OR underag* [tiab] OR youth* [tiab]))

- **CINAHL**

“health literacy” AND (adolescen* OR child* OR infant* OR juvenil* OR kids* OR paediat* OR pediat* OR school* OR student* OR teen* OR youth*)

- **PsycINFO**

(“health literacy”) AND (“adolescent” OR child* OR “minors” OR pediat* OR paediat* OR “puberty” OR school* OR student* OR boy* OR girl* OR infant* OR juvenil* OR kids* OR teenage* OR kindergar* OR teen* OR youth*)

- **Scopus**

(“health literacy”) AND (“adolescent” OR child* OR “minors” OR pediat* OR paediat* OR “puberty” OR school* OR student* OR boy* OR girl* OR infant* OR juvenil* OR kids* OR teenage* OR kindergar* OR teen* OR youth*)

### 2.2. Eligibility Criteria 

As outlined above, we focused on observational studies only. Studies were regarded as eligible if they investigated associations between health literacy and any of the eight outcome categories defined by Sørensen et al. [22]. The outcomes of the studies included were then classified into the outcome categories defined by Sørensen. Studies were excluded if they assessed health literacy with a specific focus (e.g., mental health literacy) or if they considered exclusively adult populations. The corresponding inclusion and exclusion criteria are shown in Table 1.

### 2.3. Study Selection, Data Extraction and Analysis

Extracted records were exported to reference management software. After removing duplicates, study selection was performed in three steps. First, articles were systematically selected by their titles based on inclusion criteria. Second, abstracts were screened and either included or excluded for the next screening step. Third, full texts of the remaining scientific publications were reviewed to determine whether they could be included. Two researchers independently conducted each screening step. A consensus meeting was undertaken at the end of each screening step to discuss discordances concerning the inclusion of studies. 

### 2.4. Quality Assessment

For methodological quality assessment, the Downs and Black checklist for observational studies and RCTs was applied [49]. The tool is suitable for reviews because it is designed for rating different study designs, including observational studies. To our knowledge, no suitable rating instrument for observational studies exists to date. The tool includes 27 criteria for quality assessment. All but two items can be rated with 0 or 1 points. Question 5 can be rated with 0, 1, or 2 points, and question 27 can receive 0 to 5 points so that the maximum score is 32 points. Due to the fact that only observational studies were included, 11 categories did not apply to this study design and were rated with 0 points. Accordingly, studies could score a maximum of 17 points. Two researchers independently rated and then compared study quality. 

## 3. Results

### 3.1. Literature Search

**Figure 1:**
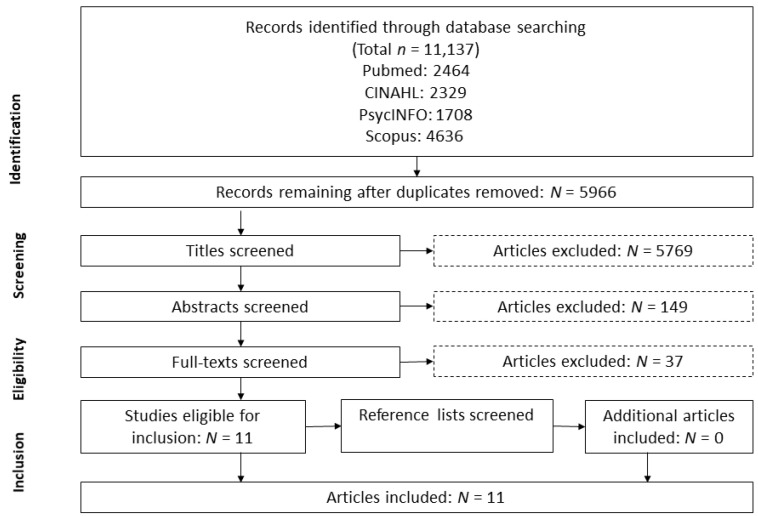
Screening process.

Across all electronic databases, the search yielded 11,137 results. After removing 5171 duplicates, 5966 studies remained for the screening process. In the first screening step, 5769 were excluded. Abstract screening of the remaining 197 studies excluded all but 48 studies. A final assessment was undertaken by screening 48 full texts. Ultimately, 11 publications met the eligibility criteria and were included for further analysis (Figure 1). The additional search of evidence published between July and November 2021 did not reveal any eligible studies.

### 3.2. Study Characteristics

The majority of studies (7 out of 11) were cross-sectional studies. All articles were published between 2011 and 2020 (Table 2) in English language, and all but 2 studies were undertaken in the USA. The studies were invariably carried out in industrialised countries. Sample size varied from 20 to 390 participants. In sum, data of 1618 children and adolescents were collected. Across all participants, ages ranged from 10 to 30 years. Three studies did not differentiate between adolescents and young adults so that the results could not be attributed to a specific age cohort. Overall, 2 studies included participants up to 24 years, and one study had participants up to 30 years without distinguishing between adults and adolescents. Studies considered different types of chronic diseases. Two publications each focused on pediatric patients with HIV, asthma, and chronic kidney disease. All other conditions (CF, hypertension, psychosocial problems, obesity, diabetes, liver disease with need for transplantation) were taken into consideration in single studies. All 11 studies found aimed to examine the significance of health literacy within chronically ill pediatric patients. The majority of the studies (9 out of 11) investigated more than one outcome category on the basis of the outcome classification by Sørensen et al. Seven studies assessed associations with health behavior and 4 studies with health service use, while 8 studies considered health outcomes. Empowerment was regarded in 5 studies. Outcomes concerning health behavior included self-management (3 studies) and medication adherence (4 studies). Health service use comprised the outcomes of emergency department visits and hospitalization (4 studies) as well as medical care received (1 study). Health outcomes included physiological parameters indicating disease progression (5 studies), morbidity (3 studies), and HRQoL (2 studies). Self-efficacy (3 studies) and transition readiness (2 studies) can be regarded as empowerment outcomes. 

### 3.3. Study Quality 

Quality scores according to the checklist by Downs and Black [49] ranged from 8.5 to 17 points (Table 3, also see Appendix A, Table A2). No study was excluded based on the quality assessment. In terms of reporting quality, the main information was provided in the majority of studies. Half of the studies did not clearly describe potential confounders. Additionally, most of the studies lacked external validity due to limited representativeness of the sample because some studies did not describe participant selection [49]. Due to missing information about the recruitment of participants, selection bias cannot be ruled out. Studies also varied with respect to sample sizes. Overall, five studies included over 100 participants. However, two studies included 20 children and adolescents as subsamples only and thus potentially lack representativeness. Additionally, the lack of differentiation in age groups may reduce the quality of analysis. All studies used appropriate tests to assess the main outcome. 

### 3.4. Health Literacy Measures

A total of seven standardized instruments on health literacy were used within the included studies. The most common tools were the Test of Functional Health Literacy in Adults (TOFHLA) [61,62] (3 studies) and the Newest Vital Sign (NVS) instrument [63] (3 studies), followed by the three-item Brief Health Literacy Screen (BHLS) [64] (2 studies) and s-TOFHLA, a short form of the TOFHLA [62,65] (2 studies). A shortened version of the European Health Literacy Survey Questionnaire (HLS-EU-Q16) [66], the Rapid Estimate of Adult Literacy in Medicine (REALM-Teen) [67], and TOFHLA-R [62] were used within 1 study each. Two studies applied more than one tool to measure health literacy. All instruments were validated and consistent, but some of them have not yet been tested in pediatric samples. That includes the BHLS, for which studies indicate high reliability in adult populations [64,68,69]. The s-TOFHLA and its original version, the TOFHLA, show high internal consistency (Cronbach’s alpha = 0.97 and 0.94) but have not yet been validated in pediatric populations [62]. The TOFHLA-R is one of two subscales of the TOFHLA and consists of a 50-item reading comprehension section [62]. The REALM-Teen is a validated instrument for adolescents and exhibits excellent internal consistency (Cronbach’s alpha = 0.94) and strong test-retest reliability [67]. The HLS-EU-Q16 was validated in people ≥15 years [66]. However, the study included participants aged 13 and 14 years [52]. Moreover, the HLS-EU-Q16 showed good reliability (Cronbach’s alpha = 0.67−0.94) and acceptable test-retest stability (rs = 0.45 to 0.90) [66]. The NVS is 64% specific and 100% sensitive for detecting inadequate health literacy [63].

### 3.5. Health Literacy and Health Behavior, Health Service Use, Health Outcomes, and Empowerment

Overall, 6 of the 11 studies described significant correlations between health literacy and considered outcomes comprising four of eight categories designed by Sørensen et al. (Table 4) [52,55,57,58,59,60]. Regarding health behavior, one of three studies on health behaviour had data showing significant associations between health literacy and self-management in an underaged population with chronic kidney disease. None of the studies showed a significant association between health literacy and treatment adherence. 

Regarding health service use, one of four studies identified significantly lower frequencies of emergency room visits and days hospitalized in pediatric patients with higher levels of health literacy. Additionally, Murphy et al. [55] found that the likelihood of receiving adequate medical care was significantly higher in populations with higher health literacy scores.

Of eight studies assessing disease status and disease course as health outcomes, five considered clinical characteristics. One of these found a significant positive correlation between health literacy and body mass index (BMI). None of the other four studies considering physiological parameters (glycemic control, viral load of HIV, CD4 cells, liver values) found a significant association with health literacy. Three studies focused on morbidity as an outcome. They found no correlation between asthma morbidity and health literacy. One study revealed that asthma patients with higher levels of health literacy reported higher frequencies of symptom days and higher rates of symptom bother. Another study identified a lower need for antibiotics in CF patients with high health literacy levels. In terms of effects on HRQoL, both studies showed significant associations between health literacy and HRQoL: One in a population of asthma patients, the other in CF patients. According to the longitudinal study carried out by Beukema et al. [50], poor health literacy was associated with a higher rate of psychosocial problems in children and adolescents suffering from psychosocial conditions, like emotional, behavioral, and social problems. However, in populations with different levels of health literacy, the level of psychosocial problems decreased at a similar rate over the course of treatment. Of the studies regarding empowerment as an outcome, two of three studies detected a significant association between health literacy and self-efficacy in populations with childhood asthma and obesity. Transition readiness was significantly associated with health literacy in one of two studies. 

## 4. Discussion

This review summarizes the published evidence on the association between health literacy and outcomes from the eight outcome categories by Sørensen et al. in pediatric patients with chronic diseases. Overall, studies provided information on four of these outcome categories, specifically health behavior, health service use, health outcomes, and empowerment. To our knowledge, this is the first systematic review on health literacy and its outcomes in chronically ill pediatric patients. While for adult populations, ample evidence is available, largely confirming associations with outcomes, a very limited number of studies was found in pediatric settings. The small number of 11 eligible studies on this topic and the partly inconsistent results reflect a lack of evidence in this field. All in all, this review found that only about half of the studies included established significant associations between health literacy and health behavior, health care use, health outcomes, and empowerment in pediatric patients. However, consistent with previous research on parental health literacy in pediatric populations [44], our results indicate a positive correlation with many of the considered outcomes.

### 4.1. Health Literacy and Health Behavior

Regarding health behavior, an association between health literacy and self-management was found in only one of three studies. None of the studies on adherence found a significant impact of health literacy in pediatric patients. This is in line with previous studies in adult populations, which did not show strong evidence with regard to health literacy and treatment adherence [70,71]. This might be explained by low variability in health literacy scores in some studies [51,72], which may conceal potential associations. Moreover, the majority of studies evaluated adherence by self-report [50,55,56], which is likely to cause bias. Quantifying adherence as an outcome (e.g., by measuring medication levels) may provide more reliable findings. Above all, additional constructs, such as positive outcome expectancy, seem to govern the level of adherence in pediatrics [56], but its relationship to health literacy is still unclear.

### 4.2. Health Literacy and Health Service Use

Health literacy showed significant associations with health service use in two of five studies. Murphy et al. [55] identified significantly improved access to the health care system. This implies, in turn, that monitoring pediatric patients with low health literacy at close intervals could be important to ensure adequate health service use and early detection of potential complications. Additionally, pediatric patients with low health literacy may benefit more from appropriate programs on health education in chronic diseases, which can lead to improved knowledge, autonomy, and patient empowerment [73]. However, parental health literacy can be assumed to strongly influence children’s health service use.

### 4.3. Health Literacy and Health Outcomes

Overall, studies on associations between health literacy and health outcomes, including physiological parameters, morbidity, and HRQoL, showed mixed results. Contrary to findings on the influence of parental health literacy on, for example, child blood glucose levels [42,43], no significant association with physiological parameters was identified for pediatric patients’ health literacy [54]. Dore-Stites et al. described parental health literacy having a stronger impact on health outcomes, such as liver function, when compared to children’s health literacy [51]. This is in line with previous research pointing out that a low level of parental health literacy is disadvantageous for child health outcomes [39,44] since it is associated with incorrect use and dosage of medication [74]. While Dore-Stites et al. [51] found no significant association between health literacy and physiological parameters, Sharif et al. [57] confirmed significant associations with the body mass index (BMI) as a parameter for obesity. Associations between health literacy and obesity have been confirmed by previous reviews in adult populations [75]. While Jackson et al. [52] could not support this association on physiological parameters, they showed a significantly less frequent need for oral antibiotics in CF patients. However, Jackson et al. [52] as well as two further studies [55,56] did not distinguish young adults up to 30 years from pediatric patients. Age-related developmental differences therefore may have remained undetected. This has to be considered because age may independently impact health behavior. Regarding the age of participants, it is striking that no study included children younger than ten years despite the fact that health literacy can be assumed to already play an important role in young children with chronic diseases. Besides, previous studies as well as those included have outlined that limited health literacy is associated with younger age [53,57,58,76]. This highlights the need for further research in younger populations. Regarding HRQoL, both studies identified significant associations, which is in line with previous findings in adult populations [77].

### 4.4. Health Literacy and Empowerment

Only two of three studies found an association with self-efficacy. A reason for these less conclusive results compared to adult populations may be the influence of puberty on levels of self-efficacy and self-management due to reduced motivation and reduced overall self-efficacy [31].

### 4.5. Study Quality and Instruments Measuring Health Literacy

Although we cannot identify significant associations between health literacy and some of the considered outcome measures, none of the study results suggest an adverse correlation. Moreover, significant results tended to be found in studies with larger sample sizes rather than those with smaller ones, indicating a potential lack of statistical power in some studies. Overall, four of five studies with more than 100 participants show significant results in terms of at least one considered outcome. Four of the five studies that did not identify any association are the ones with the smallest sample sizes. 

Overall, the identified studies used a large variety of health literacy instruments, of which only some were designed for or validated in pediatric populations [78,79], specifically the REALM-Teen and NVS. Additionally, some instruments focused on the subdimensions of health literacy, e.g., reading comprehension [63,64,67,80] and numeracy [63]. The use of different tools results in different thresholds of health literacy levels, which limits the comparability of studies. One reason for the heterogeneity of the applied health literacy measures is the lack of a commonly used definition of health literacy [81] as well as of substantial validation studies in pediatric populations. 

### 4.6. Strengths and Limitations

The results need to be considered in light of the strengths and limitations of the study. First, our literature search term was extensive in terms of the outcomes observed. Hence, the studies included are quite heterogenous in terms of diseases and outcomes examined. No meta-analysis was undertaken due to the insufficient number of studies measuring similar outcomes in comparable populations. Thus, generalisability remains debatable. However, our aim was to provide a comprehensive overview of this still-developing field of research. Due to the broad search term, we hope to have been able to include all available evidence on the impact of health literacy on outcomes. Considering that we searched four large databases that yielded 11,137 results, we assume that we were able to include the relevant studies. Second, no suitable tool for evaluating exclusively observational studies was available. Hence, we had to choose a mixed tool for RCTs and observational studies whose design made it impossible for any of the included studies to reach full scores. Nevertheless, this validated tool allowed examining diverse aspects related to study quality. Third, exclusively observational studies were examined. This entailed an overall reduced study quality because observational studies are of moderate evidence level in general. The majority of the studies were cross-sectional studies, which looked at the current state of patients rather than their disease process. Any long-term benefits from a high level of health literacy in childhood and adolescence were therefore neglected. Nevertheless, it was important to undertake this selection because we wanted to focus on the fundamental association between health literacy and outcomes in pediatric samples. The evidence found can then contribute to the design of interventions to improve health literacy in either parents or patients themselves or both. 

## 5. Conclusions

The present review highlights the fact that health literacy may play a considerable role in health behavior, health service use, and health outcomes, including in pediatric patients with chronic diseases. However, it emphasises that the current state of research is limited, and future research is needed to better understand the association between health literacy and outcomes in pediatric patients with chronic diseases. The included studies partly lacked methodological quality, warranting future studies to determine appropriate study designs and measurement instruments. In general, future studies with longitudinal designs are needed. A precondition for valid results of those studies is the implementation of reliable health literacy tools validated for underage populations. While we are aware of the challenges associated with survey research in young children, future studies should moreover focus on age-related differences concerning health literacy, including in young children. Moreover, the impact of parental health literacy on child health literacy should be examined. Additionally, key dimensions in terms of healthy literacy in children and adolescents have to be identified in order to shape and adjust the content of educational programs. Several interventional studies have been recently undertaken to improve children´s health literacy and suggest promising effects on health outcomes [38,46,82]. 

### Implications for Research and Practice

Most of the included studies used a cross-sectional design. Accordingly, complication rates, long-term prognosis, as well as psychosocial and cognitive long-term development were underrepresented outcomes. To build a profound evidence base on health literacy in pediatric patients with chronic diseases, these aspects need to be considered, requiring longitudinal studies.

All in all, the small number of identified studies in our review reflects the fact that few existing studies have focused on the children´s perspective. In contrast, the wider literature outside the scope of this review shows that caregivers and parents have frequently served as research subjects [24]. Prior research has identified significant associations between health literacy and health outcomes in adults [24]. In child and adolescent populations, research has highlighted a positive effect of education (including health literacy training) on motivation and knowledge [44]. Consequently, it seems plausible that there is a benefit in educating children themselves towards better health literacy. Moreover, in light of the fact that many chronically ill children and adolescents have low health literacy and that the prevalence of chronic diseases will further increase, interventions on the subject are an important field of action [2,83]. While growing up, children and adolescents are increasingly involved in decisions on their lives, including those related to health care. Children and adolescents with chronic conditions in particular must take over responsibility for their health and participate in their treatment. Moreover, they must be enabled to progressively deal with their diseases in everyday life, at the latest by the time they reach adulthood. For this purpose, health literacy may provide a well-founded base for making adequate decisions and taking over responsibility for one´s own health [22]. The influence of developmental stages, different ages, and particularly puberty on health literacy and its associations with outcomes should be considered in future research, for it may impact health behavior. Moreover, on the basis of sound data on the relevance of health literacy, future research should focus on the practical implementation of health literacy. 

Recapitulating the research process, it is striking that the studies included provided information on only four of eight possible outcome categories. The influence of health literacy on sustainability, equity, participation, and health costs were therefore neglected in this review. One reason for this may be the aim of the study, focusing on the individual perspective of children and adolescents. However, future research should contemplate the remaining aspects in order to depict health literacy comprehensively. Furthermore, in order to embed health literacy into a larger context, future research should examine the impact of environmental factors on adolescents´ health literacy. In consideration of all these contextual factors, research may be able to provide politics with recommendations on how to create an appropriate setting in which children and adolescents with chronic conditions can develop unrestrained.

## Figures and Tables

**Table 1:** Eligibility criteria.

Criterion	Inclusion	Exclusion
Population	Sample or subsample composed of children and adolescents under the age of 18 years diagnosed with a chronic disease Samples that included the defined age group but additionally included patients up to 30 years of age without differentiating their analysis by age groups were included as well	Sample composed of persons aged 18 years and older only Healthy children and adolescents Acute diseases
Focus/outcomes	Impact of health literacy in children and adolescents affected by chronic diseases on outcome categories defined by Sørensen et al. (health behavior, health outcomes, health service use, empowerment, participation, health costs, equity, and sustainability)	Health literacy of parents or caregivers Health literacy in children without chronic diseases
Study design	Observational studies	Interventional studies Meta-analyses Systematic reviews Randomized controlled trials Qualitative studies Case reports Expert opinions
Language	English, German	Studies in any language other than English or German

**Table 2:** Study characteristics.

Author	Publication Year	Country	Study Design	Sample Size	Median Age of Participants (Range)	Health Issues
**Beukema et al. [50]**	2019	Netherlands	Prospective cohort study	390	15 (12–18)	Psychosocial/mental diseases
**Dore-Stites et al. [51]**	2019	USA	Cross-sectional study	Total: 79 Subgroup: 20	Total: N/A (N/A) Subgroup: N/A (13–18)	Liver transplant recipients
**Jackson et al. [52]**	2019	Ireland	Retrospective cohort study	251	21.38 (13–30)	Cystic fibrosis
**Levine et al. [53]**	2018	USA	Retrospective cohort study	49	N/A (12–18)	Chronic kidney diseases, SLE, kidney transplant, dialysis
**Manegold et al. [54]**	2019	USA	Cross-sectional study	65	15.03 (13–17)	Diabetes mellitus type 1
**Murphy et al. [55]**	2010	USA	Longitudinal cohort study	186	20.5 (16–24)	HIV
**Navarra et al. [56]**	2014	USA	Cross-sectional study	50	19.7 (13–24)	HIV
**Sharif et al. [57]**	2011	USA	Cross-sectional study	78	11.5 (10–16)	Obesity
**Valerio et al. [58]**	2016	USA	Cross-sectional study	181	N/A (15–19)	Asthma
**Valerio et al. [59]**	2018	USA	Cross-sectional study	327	15.8 (13–18)	Asthma
**Zhong et al. [60]**	2020	USA	Cross-sectional study	Total: 59 Subgroup: 21	Total: N/A (12–29) Subgroup: N/A (12–18)	Chronic kidney disease, hypertension

USA, United States of America; N/A, not available; SLE, systematic lupus erythematosus; HIV, human immunodeficiency virus.

**Table 3:** Quality assessment.

Criterion	Beukema et al. (2019)	Dore-Stites et al. (2019)	Jackson et al. (2019)	Levine et al. (2018)	Manegold et al. (2019)	Murphy et al. (2010)	Navarra et al. (2014)	Sharif et al. (2011)	Valerio et al. (2016)	Valerio et al. (2018)	Zhong et al. (2020)
Total	17	9.5	11.5	9.5	9	12	12	12.5	11	8.5	12.5

**Table 4:** Influence of health literacy on health outcomes.

Outcome	Regarded in *n* Studies	Study	Negative Correlation	Positive Correlation (*p*)
**Health behavior**	**7**			
Adherence	4	Beukema et al.	No	Yes (0.23)
		Dore-Stites et al.	N/A	N/A
		Murphy et al.	No	Yes (0.98)
		Navarra et al. TOFHLA REALM-Teen	No No	Yes (0.94) Yes (0.40)
Self-management/care	3	Beukema et al.	No	Yes (0.16)
		Valerio et al., 2016 Rescue medication Controller medication	No No	Yes (0.774) Yes (0.447)
		Zhong et al.	No	**Yes (0.05)**
**Health service use**	**4**			
Emergency department (ED) visits/hospitalization/hospital stays	4	Jackson et al. Outpatient visits Days hospitalized	No No	Yes (0.432) Yes (0.329)
		Levine et al.	N/A	N/A
		Murphy et al. ED visits: ≥1 (ref. none) Overnight hospital stays: ≥1 (ref. none)	No No	Yes (0.28) Yes (0.14)
		Valerio et al., 2016	No	**Yes (0.003)**
Medical care received	1	Murphy et al.	No	**Yes (0.0002)**
**Health outcomes**	**8**			
Physiological parameters/clinical characteristics	5	Dore-Stites et al.	N/A	N/A
		Jackson et al. ppFEV1 BMI Pseudomonas aeruginosa Number of iv antibiotics Duration of iv antibiotics Number of oral antibiotics Duration of oral antibiotics	No No No No No No No	Yes (0.763) Yes (0.649) Yes ((0.649 Yes (0.329 Yes (0.295) **Yes (0.004)** **Yes (0.004)**
		Manegold et al. Glycemic control	No	Yes (0.43)
		Murphy et al. CD4 cells Viral load	No No	Yes (0.15) Yes (0.13)
		Sharif et al.	No	**Yes (<0.0001)**
Morbidity	3	Beukema et al.	No	**Yes (0.001)**
		Valerio et al., 2016	No	Yes (0.404)
		Valerio et al., 2018 Symptom bother Symptom days	No No	**Yes (0.05)** Yes (0.16)
HRQoL	2	Jackson et al.	No	**Yes (0.004)**
		Valerio et al., 2016	No	**Yes (0.016)**
**Empowerment**	**5**			
Self-efficacy	3	Murphy et al. Adherence to medication regimes Keeping of medical appointments	No No	Yes (0.55) Yes (0.85)
		Sharif et al.	No	**Yes (<0.0001)**
		Valerio et al., 2018	No	**Yes (0.02)**
Transition readiness	2	Manegold et al.	No	Yes (0.50)
		Zhong et al.	No	**Yes (0.001)**

N/A, not available; HL, health literacy; ED, emergency department; ppFEV1, percent predicted forced expiratory pressure in 1 s; BMI, body mass index; iv, intravenous; HRQoL, Health-Related Quality of Life; bold text indicates statistically significant results.

## Appendix A

**Table A1:** Study characteristics.

Author	Publication Year	Country	Study Design	Sample Size	Median Age of Participants (Range)	Health Issues	Health Literature Measure	Outcomes
**Beukema et al. [50]**	2019	Netherlands	Prospective cohort study	390	15 (12–18)	Psychosocial/mental diseases	3-item HL Screening questions	Adherence Learning process (improved understanding) Confidence Psychosocial outcomes (SDQ)
**Dore-Stites et al. [51]**	2019	USA	Cross-sectional study	Total: 79 Subgroup: 20	Total: N/A (N/A) Subgroup: N/A (13–18)	Liver transplant recipients	TOFHLA Newest vital sign	Adherence (tacrolimus blood level) Liver function (AST, ALT, TBili)
**Jackson et al. [52]**	2019	Ireland	Retrospective cohort study	251	21.38 (13–30)	Cystic fibrosis	HLS-EU-Q16	HRQoL (CFQr) BMI, ppFEV1, *P. aeruginosa* Days of iv/oral antibiotics Outpatient visits Days hospitalized
**Levine et al. [53]**	2018	USA	Retrospective cohort study	49	N/A (12–18)	Chronic kidney diseases, SLE, kidney transplant, dialysis	Newest vital sign	ED visits Preventable hospitalizations Total hospitalizations Total inpatient days
**Manegold et al. [54]**	2019	USA	Cross-sectional study	65	15.03 (13–17)	Diabetes mellitus type 1	TOFHLA-R	Transition readiness (TRAQ) Glycemic control (Hba1c)
**Murphy et al. [55]**	2010	USA	Longitudinal cohort study	186	20.5 (16–24)	HIV	TOFHLA STOFHLA	Viral load, CD4 cells Self-efficacy for adherence to HIV medication regimes/keeping of medical appointments Medical care received (diabetic self-care practice instrument (adopted for HIV+ patients) and Module 1 of the Pediatric Adherence Questionnaire and Self-efficacy for health promotion and risk reduction) ED visits Overnight hospital stays
**Navarra et al. [56]**	2014	USA	Cross-sectional study	50	19.7 (13–24)	HIV	TOFHLA REALM-Teen	Adherence to ART (BAMS)
**Sharif et al. [57]**	2011	USA	Cross-sectional study	78	11.5 (10–16)	Obesity	STOFHLA	BMI Eating self-efficacy (ESES Questionnaire)
**Valerio et al. [58]**	2016	USA	Cross-sectional study	181	N/A (15–19)	Asthma	3-item HL screening questions	HLQoL (MiniPAQLQ) Asthma management: medication use Morbidity (EPRII guidelines) Hospitalization
**Valerio et al. [59]**	2018	USA	Cross-sectional study	327	15.8 (13–18)	Asthma	REALM-Teen	Asthma self efficacy Morbidity (symptom days, symptom bother)
**Zhong et al. [60]**	2020	USA	Cross-sectional study	Total: 59 Subgroup: 21	Total: N/A (12–29) Subgroup: N/A (12–18)	Chronic kidney disease, hypertension	Newest vital sign	HCT readiness (STAR_x_ Questionnaire) Self-management

N/A, not available; HL, Health Literaca; ART, Anti-Retroviral Therapy; HCT, Health Care Transition; ED, Emergency Department; SDQ, Dutch self-reported and parent-reported versions of Strengths and Difficulties Questionnaire; TOFHLA, Test of Functional Health Literacy in Adults; REALM-Teen, Rapid Estimate of Adult Literacy in Medicine; BAMS, Beliefs About Medication Scale; ESES, Eating Self-Efficacy Scale; MiniPAQLQ, Mini Pediatric Asthma Quality of Life Questionnaire; STAR_x_, Self-Management and Transition to Adulthood with R_x_ treatment questionnaire.

**Table A2:** Quality assessment.

Criterion	Beukema et al. (2019)	Dore-Stites et al. (2019)	Jackson et al. (2019)	Levine et al. (2018)	Manegold et al. (2019)	Murphy et al. (2010)	Navarra et al. (2014)	Sharif et al. (2011)	Valerio et al. (2016)	Valerio et al. (2018)	Zhong et al. (2020)
	**Reporting**										
1: Hypothesis/aim/objective clearly described	1	1	1	1	1	0.5	1	1	1	1	1
2: Main outcomes in introduction or methods	1	1	1	1	1	0.5	1	1	1	1	1
3: Patient characteristics clearly described	1	1	1	1	1	1	1	1	1	1	1
4: Interventions of interest clearly described	0	0	0	0	0	0	0	0	0	0	0
5: Principal confounders clearly described	2	0	0	2	0	1	2	2	0	0	2
6: Main findings clearly described	1	1	1	0.5	1	1	1	1	1	0.5	1
7: Estimates of random variability provided for main outcomes	1	1	1	0	1	0.5	1	1	1	0.5	1
8: All adverse events of intervention reported	0	0	0	0	0	0	0	0	0	0	0
9: Characteristics of patients lost to follow-up described	1	0	0	0	0	1	0	0	0	0	0
10: Probability values reported for main outcomes	1	1	1	0	1	1	1	1	1	1	1
	**External validity**										
11: Subjects asked to participate were representative of source population	1	0	1	0	0	0	0	0	1	0.5	0
12: Subjects prepared to participate were representative of source population	1	0.5	0.5	0	0	0.5	0	0.5	1	0	0.5
13: Location and delivery of study treatment was representative of source population	0	0	0	0	0	0	0	0	0	0	0
	**Internal validity—bias and confounding**										
14: Study participants blinded to treatment	0	0	0	0	0	0	0	0	0	0	0
15: Blinded outcome assessment	0	0	0	0	0	0	0	0	0	0	0
16: Any data dredging clearly described	1	1	1	1	1	1	1	1	1	1	1
17: Analyses adjust for differing lengths of follow-up	1	0	0	0	0	0	0	0	0	0	0
18: Appropriate statistical tests performed	1	1	1	1	1	1	1	1	1	1	1
19: Compliance with interventions was reliable	0	0	0	0	0	0	0	0	0	0	0
20: Outcome measures were reliable and valid	1	1	1	1	1	1	1	1	1	1	1
21: All participants recruited from the same source population	0	0	0	0	0	0	0	0	0	0	0
22: All participants recruited over the same period	0	0	0	0	0	0	0	0	0	0	0
23: Participants randomized to treatment(s)	0	0	0	0	0	0	0	0	0	0	0
24: Allocation of treatment concealed from investigators and participants	0	0	0	0	0	0	0	0	0	0	0
25: Adequate adjustment for confounding	1	0	1	1	0	1	1	1	0	0	1
26: Losses to follow-up taken into account	1	0	0	0	0	1	0	0	0	0	0
	**Power**										
27: Sufficient power to detect treatment effect at significance	0	0	0	0	0	0	0	0	0	0	0
**Total**	**17**	**9.5**	**11.5**	**9.5**	**9**	**12**	**12**	**12.5**	**11**	**8.5**	**12.5**

**Table A3:** Influence of health literacy on outcomes.

Outcome	Regarded in *n* Studies	Positive Correlation	Reported Estimate	95% CI	*p*
**Health behavior**	**7**				
Adherence	4	Beukema et al.	0.43 (β coefficient)	−0.27, 1.14	0.23
		Dore-Stites et al.	N/A	N/A	N/A
		Murphy et al. ≥90% adherence (ref. 0%) >0% and <90% (ref. 0%)	1.00 (odds ratio) 1.00 (odds ratio)	(0.96–1.05) (0.95–1.04)	0.98
		Navarra et al. TOFHLA REALM-Teen	−0.011 (correlation coefficient) −0.122 (correlation coefficient)	N/A N/A	0.94 0.40
Self-management/care	3	Beukema et al. Improved understanding	0.37 (β coefficient)	−0.15, 0.90	0.16
		Valerio et al., 2016 Rescue medication Controller medication	1.12 (odds ratio) 1.33 (odds ratio)	0.53, 2.34 0.64, 2.80	0.774 0.447
		Zhong et al. Adequate HL (ref. Low/moderate HL)	N/A	N/A	**0.05**
**Health service use**	**4**				
ED visits/hospitalization/hospital stays	4	Jackson et al. Outpatient visits Days hospitalized	−0.06 (correlation coefficient) −0.08 (correlation coefficient)	−0.18–0.07 −0.2–0.05	0.432 0.329
		Levine et al. ED visits Preventable hospitalizations Total hospitalizations Total number of days inpatient	N/A	N/A	N/A
		Murphy et al. ED visits: ≥1 (ref. none) Overnight hospital stays: ≥1 (ref. none)	0.98 (odds ratio) 0.97 (odds ratio)	0.96–1.01 0.93–1.01	0.28 0.14
		Valerio et al., 2016 Hospitalization and inadequate HL	1.37 (odds ratio)	1.11, 1.69	**0.003**
Medical care received	1	Murphy et al. Medical care received ≥3 times (ref. 0) 1–2 times (ref. 0)	1.09 (odds ratio) 1.06 (odds ratio)	1.04–1.15 1.02–1.09	**0.0002**
**Health outcomes**	**8**				
Physiological parameters/clinical characteristics	5	Dore-Stites et al. AST, ALT, TBili	N/A	N/A	N/A
		Jackson et al. ppFEV1 Body mass index Pseudomonas aeruginosa Number of iv antibiotics Duration of iv antibiotics Number of oral antibiotics Duration of oral antibiotics	−0.02 (correlation coefficient) 0.03 (correlation coefficient) −0.03 (correlation coefficient) −0.08 (correlation coefficient) −0.08 (correlation coefficient) −0.21 (correlation coefficient) −0.23 (correlation coefficient)	−0.14–0.11 −0.09–0.15 −0.16–0.09 −0.2–0.05 −0.21–0.04 −0.33–−0.09 −0.34–−0.11	0.763 0.649 0.649 0.329 0.295 **0.004** **0.004**
		Manegold et al. Glycemic control	−0.05 (correlation coefficient)	N/A	0.43
		Murphy et al. CD4 cells Viral load	N/A N/A	N/A N/A	0.15 0.13
		Sharif et al. Body mass index	−0.016 (correlation coefficient) −0.43 (β coefficient)	−0.025, −0.008	**<0.0001**
Morbidity	3	Beukema et al. Psychosocial problems	−1.70 (β coefficient)	−2.72, −0.69	**0.001**
		Valerio et al., 2016 Moderate–severe asthma	1.35 (odds ratio)	0.67, 2.71	0.404
		Valerio et al., 2018 Symptom bother Symptom days	0.95 (β coefficient) 0.23 (β coefficient)	0.005, 1.89 −0.09, 0.55	**0.05** 0.16
HRQoL	2	Jackson et al.	0.23 (correlation coefficient)	0.11–0.35	**0.004**
		Valerio et al., 2016	0.75 (odds ratio)	0.59, 0.95	**0.016**
**Empowerment**	**5**				
Self-efficacy	3	Murphy et al. To adhere to medication regimens: mean ≥4 (ref. mean <4) To keep medical appointments: mean ≥4 (ref. mean <4)	0.99 (odds ratio) 1.01 (odds ratio)	0.95–1.03 0.95–1.06	0.55 0.85
		Sharif et al.	−0.45	N/A	**<0.0001**
		Valerio et al., 2018	1.28 (β coefficient)	0.23, 2.32	**0.02**
Transition readiness	2	Manegold et al.	−0.10 (correlation coefficient)		0.50
		Zhong et al. Adequate HL (ref. Low/moderate HL)	N/A	N/A	**0.001**

N/A, not available; HL, health literacy; ED, emergency department; ppFEV1, percent predicted forced expiratory pressure in 1 s; iv, intravenous; bold text indicates statistically significant results.
